# Monte Carlo investigation of energy response of various detector materials in I125 and Yb169 brachytherapy dosimetry

**DOI:** 10.1120/jacmp.v11i4.3282

**Published:** 2010-07-28

**Authors:** T. Palani Selvam, Biju Keshavkumar

**Affiliations:** ^1^ Radiological Physics and Advisory Division Health, Safety and Environment Group, Bhabha Atomic Research Centre Mumbai India

**Keywords:** brachytherapy, Monte Carlo, energy response, phantoms

## Abstract

Relative absorbed‐dose energy response correction R for different detector materials in water, PMMA and polystyrene phantoms are calculated using Monte Carlo‐based EGSnrc code system for I125 and Yb169 brachytherapy sources. The values of R obtained for I125 source are 1.41, 0.92, 3.97, 0.47, 8.32 and 1.10, respectively, for detector materials LiF, ${\text{Li}}_{2}B_{4}O_{7},\;{\text{Al}}_{2}O_{3}$, diamond, silicon diode and air. These values are insensitive to source‐to‐detector distance and phantom material. For Yb169 source, R is sensitive to source‐to‐detector distance for detector materials other than air and ${\text{Li}}_{2}B_{4}O_{7}$. For silicon, R increases from 3 to 4.23 when depth in water is increased from 0.5 cm to 15 cm. For Yb169 source, the values of R obtained for air and ${\text{Li}}_{2}B_{4}O_{7}$ in PMMA and polystyrene phantoms are comparable to that obtained in water. However, LiF, Si and ${\text{Al}}_{2}O_{3}$ show enhanced response and diamond shows decreased response in PMMA and polystyrene phantoms than in water.

PACS number: 87.53.Jw

## I. INTRODUCTION

Liquid water serves as reference medium for dosimetry of interstitial brachytherapy sources.^(^ ^1^ ^)^ Accurate dose measurements in the vicinity of individual brachytherapy sources are difficult. Ionization chambers are rarely used in brachytherapy dose measurements.^(^ ^2^ ^–^ ^5^ ^)^ This is because conventional ion chambers either lack the sensitivity to measure accurately the low levels of radiation emanating from individual sources, or are so large that dose gradients across the sensitive volume compromise spatial resolution. Solid state experimental dosimeters, such as silicon (Si) diodes, diamonds, ${\text{Al}}_{2}O_{3}$, LiF and ${\text{Li}}_{2}B_{4}O_{7}$ thermoluminescent dosimeters (TLD), provide the necessary sensitivity and spatial resolution but exhibit energy dependent responses which will vary with the source energy and with the position of the dosimeter within an absorbing medium.

Many studies are reported on dosimetric measurements around the I125,Yb169,Cs137,Pd103 and Ir192 brachytherapy sources using the solid state detectors such as LiF, ${\text{Li}}_{2}B_{4}O_{7}$, Si diode, diamond, ${\text{Al}}_{2}O_{3}$.^(^ ^6^ ^–^ ^23^ ^)^ Relative diode response which is defined as the measured detector reading per measured unit of air kerma varies by 14%, 40%, and 75% with respect to source‐detector distance in the presence of Cs137,Yb169 and Ir192 sources, respectively.^(^ ^12^ ^,^ ^14^ ^)^ TLD‐100 response has been shown to vary by 10% with respect to distance from a Ir192 source.^(^ ^9^ ^)^

Monte Carlo techniques are widely used for calculating energy response corrections because of the ease with which the 3D geometry of the phantom, detector and source can be modeled. For example, Williamson et al.,^(^ ^12^ ^)^ Piermattei et al.^(^ ^11^ ^)^ and Valicenti et al.^(^ ^16^ ^)^ employed the Monte Carlo‐based MCPT code for calculating such correction factors. MacPherson et al.^(^ ^15^ ^)^ used MCNP (version 4) Monte Carlo code to calculate absorbed‐dose energy response correction for Yb169 source. Mobit et al.^(^ ^22^ ^)^ used the EGSnrc Monte Carlo system^(^ ^24^ ^)^ to calculate the corrections for LiF TLD rods for I125 sources.

According to TG43U1 update^(^ ^1^ ^)^ for brachytherapy dose measurements a well‐characterized energy response function should be quantitatively accounted for. The objective of the present study is to calculate the relative absorbed‐dose energy response correction as a function of depth in water for different solid‐state detector materials for I125 (model selectSeed) and high‐dose rate (HDR) Yb169 (model 4140) brachytherapy sources. The study also includes air as detector material representing an ionization chamber. Measurement of dose distribution is usually performed in ‘water‐equivalent’ solid phantoms. The solid phantoms have the advantage that they can be precisely machined to accommodate sources and detectors, and distances can be accurately determined. Therefore, the study also includes calculation of relative absorbed‐dose energy response correction for the investigated detector materials in solid phantoms polystyrene and polymethyl methacrylate (PMMA). We have employed the Monte Carlo‐based DOSRZnrc and FLURZnrc user‐codes^(^ ^24^ ^)^ of the EGSnrc code system^(^ ^25^ ^)^ in the present work.

## II. MATERIALS AND METHODS

### A. Radioactive sources and detectors

The geometric details and composition of the I125 selectSeed and Yb169 model 4140 are taken from the published studies.^(^ ^26^ ^,^ ^27^ ^)^ The photon energy spectra of the I125 and Yb169 sources needed for the Monte Carlo calculations are taken from literature.^(^ ^1^ ^,^ ^27^ ^)^ The detector materials investigated in the present study are LiF, ${\text{Li}}_{2}B_{4}O_{7},\;{\text{Al}}_{2}O_{3}$, diamond, silicon and air. Table 1 presents the values of $Z_{\text{eff}}$ (effective atomic number), 〈Z/A〉 (electron density) and ρ (mass density) of the investigated detector materials.

**Table 1:** Values of effective atomic number $Z_{\text{eff},}$ electron density 〈Z/A〉, and mass density ρ of the detector materials studied.

*Detector material*	$Z_{\text{eff}}$	〈Z/A〉,	$\rho ({\text{gcm}}^{‐3})$
Water	7.5	0.555	1.0
LiF	8.27	0.462	2.635
${\text{Li}}_{2}B_{4}O_{7}$	7.4	0.485	2.44
${\text{Al}}_{2}O_{3}$	10.2	0.491	3.97
Diamond	6	0.496	3.5
Silicon	14	0.499	2.33
Air	7.6	0.499	0.0012

### B. Energy dependence of the detector

The energy dependence of the detector may be separated into two components.^(^ ^28^ ^–^ ^29^ ^,^ ^30^ ^)^ One function, called the intrinsic energy‐dependence, $k_{\text{bq}}(Q)$, relates the detector output, $M_{\text{det}}\;(Q)$, to the average dose to the material of the sensitive detector element, $D_{\text{det}}\;(Q)$, as a function of beam quality, *Q*.

(1) $$D_{\det}(Q)=k_{bq}(Q)\;M_{\det}(Q)$$

The other function, denoted the absorbed‐dose energy dependence, *f*(*Q*), relates $D_{\text{det}}\;(Q)$ to the dose to another medium, $D_{\text{med}}\;(Q)$, in the absence of the detector, as a function of *Q*.

(2) $$D_{med}(Q)=f(Q)\;D_{\det}(Q)$$

For a cavity (detector) that is large in comparison to range of electrons,

(3) $$f(Q)={\left(\frac{{\bar{\mu }}_{en}}{\rho }(Q)\right)}_{\det}^{wat}$$

where ${\left(\frac{{\bar{\mu }}_{\text{en}}}{\rho }(Q)\right)}_{\text{det}}^{\text{wat}}$ is ratio of mean mass‐energy absorption coefficient of medium‐to‐detector at Q.

The above equation is applicable when there is charged particle equilibrium and the energy fluence spectrum of photon is not perturbed by the detector. For more details on *f*(*Q*), see DeWerd et al.^(^ ^28^ ^)^

In brachytherapy, quantity of interest is dose to water. The detectors are generally calibrated against a reference beam, which is usually Co60. The relative absorbed‐dose energy response correction *R* is the largest single source of Type B (systematic) uncertainty for TLD and other secondary dosimeters used in brachytherapy dosimetry. For a given detector material and a beam quality *Q*, *R* is defined as:

(4) $$R=\frac{{\left(D_{\det}/D_{wat}\right)}_{Q}}{{\left(D_{\det}/D_{wat}\right)}_{60_{Co}}}$$

where the numerator represents detector‐to‐water dose ratio at Q(I125 or Yb169), and the denominator represents the same dose ratio at Co60.

In the presence of charged particle equilibrium and when the detector material does not alter the photon energy fluence spectrum (see Eq. (3)), the above equation can be written as:

(5) $$R=\frac{\left[1/f(Q)\right]}{\left[1/f({\;}^{60}Co)\right]}=\frac{{\left[{\left(\mu_{en}/\rho \right)}_{\det}/{\left(\mu_{en}/\rho \right)}_{wat}\right]}_{Q}}{{\left[{\left(\mu_{en}/\rho \right)}_{\det}/{\left(\mu_{en}/\rho \right)}_{wat}\right]}_{60_{Co}}}$$

Here, the numerator represents ratio of mean mass‐energy absorption coefficient of detector‐to‐water at *Q*, and the denominator represents the same ratio at Co60.

**Figure 1:**
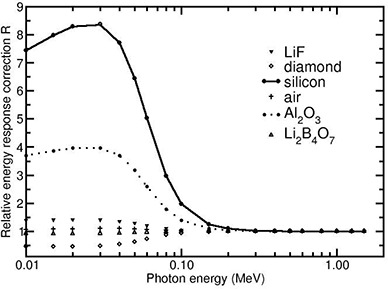
Values of the relative absorbed‐dose energy response correction R presented for different detector materials shown as a function of photon energy in the range 10 keV–1.5 MeV. The values are calculated using the mass‐energy‐absorption coefficients of the detector materials and water.

Figure 1 presents the values of *R* for the investigated detector materials shown as a function of photon energy in the range 10 keV to 1.5 MeV. The values are based on the mass energy absorption coefficients data by Hubbell and Selzter.^(^ ^31^ ^)^

### C. Monte Carlo calculations

#### C.1 DOSRZnrc simulations of dose ratios for 60Co beam

Calculation of dose ratios at Co60 is important to derive *R* (see denominator of Eq. (4)). Dose ratios in water phantom for the investigated detector materials for the Co60 beam, ${\left[D_{\text{det}}/D_{\text{wat}}\right]}_{60_{\text{Co}}}$, are calculated using the DOSRZnrc user code^(^ ^24^ ^)^ of EGSnrc code system.^(^ ^25^ ^)^ Here, $D_{\text{det}}$ and $D_{\text{wat}}$ represent dose to detector and dose to water, respectively. In the Monte Carlo calculations, a parallel Co60 beam is incident on a 20 cm radius by 40 cm height cylindrical water phantom. The beam has a radius of 5.64 cm at the front face of the phantom (field size is $100\;{\text{cm}}^{2}$). A realistic Co60 spectrum from a telecobalt unit distributed along with the EGSnrc code system^(^ ^25^ ^)^ is used in the calculations. Cylindrical detector materials of 0.5 cm diameter and varying thicknesses are positioned at a depth 0.5 cm along the central axis of the water phantom. The thicknesses of the detector material are varied from 0.1 cm to 0.5 cm to study the influence of the thickness of the detector on ${\left[D_{\text{det}}/D_{\text{wat}}\right]}_{60_{\text{Co}}}$.

#### C.2 FLURZnrc simulations of collision kerma and mean energies for 125I and 169Yb sources

For the calculation of dose ratio of detector‐to‐water for the I125 and Yb169 sources (numerator of Eq. (4)), we used the FLURZnrc user‐code.^(^ ^24^ ^)^ In the calculations, the photon fluence spectrum is scored in 0.5 mm thick and 0.5 mm high cylindrical shells, along the transverse axis of the sources (distances, 0.5 cm–15 cm) in the 20 cm radius by 40 cm high cylindrical phantoms. The fluence spectrum is converted to collision kerma to water and collision kerma to detector materials by using the mass‐energy absorption coefficients of water and detector materials.^(^ ^28^ ^)^ Using the values of collision kerma to water and collision kerma to detector materials, the denominator of Eq. (1) is obtained for the I125 and Yb169 sources. In the calculation of collision kerma to detector materials, no detector material is present. We have assumed that the presence of the detector materials does not affect the photon fluence spectrum. At I125 and Yb169 photon energies, charged particle equilibrium exists and the collision kerma may be approximated to absorbed dose.

The fluence weighted mean energy ${\bar{E}}_{f}$ and the detector‐kerma weighted mean energy ${\bar{E}}_{k,m}$ (suffix *m* represents detector material) are calculated as a function of distance from the source along the transverse axis using the following expressions:

(6) $${\bar{E}}_{f}=\frac{\int E\phi (E)dE}{\int \phi (E)dE}$$

(7) $${\bar{E}}_{k,m}=\frac{\int E^{2}\phi (E){\left({\;}^{\mu_{en}(E)}/\rho \right)}_{m}dE}{\int E\phi (E){\left({\;}^{\mu_{en}(E)}/\rho \right)}_{m}dE}$$

where *E* is the kinetic energy of photon in keV, Φ(E) is the differential photon fluence spectrum at *E* about *dE* and ${\left(\mu_{\text{en}}(E)/\rho \right)}_{m}$ is the mass energy absorption coefficient of the detector material m at the photon energy *E*. The values ${\bar{E}}_{f}$ of and ${\bar{E}}_{k,m}$ are calculated for the I125 (selectSeed) and Yb169 (model 4140) as well as for the bare sources.

#### C.3 Monte Carlo parameters and statistical uncertainties

The PEGS4 dataset needed for Monte Carlo calculations described above is based on XCOM^(^ ^32^ ^)^ compilations. We set AE=0.521MeV (kinetic energy of the electron is 0.01 MeV) and AP=0.001MeV while generating the PEGS4 dataset, where the parameters AE and AP are the low‐energy thresholds for the production of knock‐on electrons and secondary bremsstrahlung photons, respectively. All the calculations utilized the PRESTA‐II step length and EXACT boundary crossing algorithms. In all calculations, electron range rejection technique is used to save computation time. We set ESAVE=2MeV for this purpose.

The photon transport cut off energy PCUT is chosen at 1 keV in all calculations. In DOSRZnrc calculations, we set AE=ECUT=0.521MeV (10 keV kinetic energy). In the FLURZnrc calculations, electrons are not transported by setting electron transport cutoff parameter ECUT=2MeV (kinetic energy). Up to $10_{9}$ photon histories are simulated. The 1 σ statistical uncertainties on the calculated DOSRZnrc‐based dose values are generally within 0.3%. The 1σ statistical uncertainties on the calculated FLURZnrc‐based collision kerma values are usually 0.1% and never exceeded 0.2%. The statistical uncertainties on the calculated values of *R* are less than 0.6%. Throughout the text, the number shown in parentheses following a value represents the absolute uncertainty on the last digit of the value with a coverage factor k=1.

## III. RESULTS & DISCUSSION

### A. Mean energies

An analysis of XCOM data shows that the interaction mechanisms at 27 keV photons in water are 46.4% photo electric absorption, 41% Compton scattering and 12.6% coherent scattering. At this energy, even after multiple Compton scattering in water, the energy of the scattered photons does not change significantly. Hence, the mean energies of the I125 source do not change with the depth in water. For example, ${\bar{E}}_{f}$ for the selectSeed I125 source and the bare I125 line source is about 28 keV in water, independent of distance.

**Figure 2:**
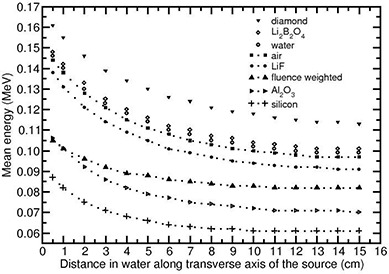
Monte Carlo‐calculated collision kerma weighted mean energies of different detector materials shown as a function of distance along transverse axis of the Yb169 source (model 4140) in water. Also shown is the fluence weighted mean energy in water for comparison purpose.

For the Yb169 source, mean energies decrease with distance in water. This is due to substantial degradation in the photon energy after scattering. Figure 2 presents the values of and for ${\bar{E}}_{k,m}$ the Yb169 (model 4140) source at various transverse axis distances in water. The energy degradation is significant in PMMA and polystyrene phantoms when compared to water phantom because scattering is high in PMMA and polystyrene.

### B. Dose ratios for 60Co beam

Table 2 presents the values of ${\left[D_{\text{det}}/D_{\text{wat}}\right]}_{60_{\text{Co}}}$ for different detector materials at 0.5 cm depth in water phantom for various detector thicknesses. Also shown in this Table are the values of ${\left[{\left(\mu_{en}/\rho \right)}_{\det}/{\left(\mu_{en}/\rho \right)}_{wat}\right]}_{60_{Co}}$ calculated at 1.25 MeV, and $<Z/A{>}_{\det}/<Z/A{>}_{wat}$, for comparison purposes. For a given detector material, the dose ratio is independent of detector thickness. It is interesting to see that the values of ${\left[{\left(\mu_{en}/\rho \right)}_{\det}/{\left(\mu_{en}/\rho \right)}_{wat}\right]}_{60_{Co}}$ and $<Z/A{>}_{\det}/<Z/A{>}_{wat}$ agree well with the values of ${\left[D_{\text{det}}/D_{\text{wat}}\right]}_{60_{\text{Co}}}$ (a maximum difference of 1.8% is observed for the air material). This suggests that at the Co60 energies, the investigated detectors (thickness from 0.1 cm to 0.5 cm) behave like a photon detector, as Compton scattering is the predominant interaction in all the detector materials. This implies that dose to detector is related to dose to water by the relation $D_{\text{det}}/D_{\text{wat}}={\left[{\left(\mu_{en}/\rho \right)}_{\det}/{\left(\mu_{en}/\rho \right)}_{wat}\right]}_{60_{Co}}$. As the difference between the values of ${\left[{\left(\mu_{en}/\rho \right)}_{\det}/{\left(\mu_{en}/\rho \right)}_{wat}\right]}_{60_{Co}}$ and ${\left[D_{\text{det}}/D_{\text{wat}}\right]}_{60_{\text{Co}}}$ is small (see Table 2), we have used ${\left[{\left(\mu_{en}/\rho \right)}_{\det}/{\left(\mu_{en}/\rho \right)}_{wat}\right]}_{60_{Co}}$ values for calculating *R*.

**Table 2:** Monte Carlo‐calculated ratio of dose to detector and dose to water for different detector materials for Co60 beam presented for different detector thickness. The number shown in parentheses following a value represents the absolute uncertainty on the last digit of the value with a coverage factor k=1. The radius of the detector is 5 mm. These detectors are at a depth of 0.5 cm in a 20 cm radius by 40 cm height unit density water phantom. The Co60 beam has a radius of 5.64 cm at the phantom surface. Also shown in this table are the values of ratio of mass‐energy‐absorption coefficients of detector to water calculated at the Co60 energy (1.25 MeV) and the values of ratio of <Z/A> of detector to water.

*Detector Material*	*Thickness of Detector (mm)*				${\left[\frac{{\left(\mu_{en}/\rho \right)}_{\det}}{{\left(\mu_{en}/\rho \right)}_{wat}}\right]}_{{\;}^{60}Co}$	$\frac{<Z/A{>}_{\det}}{<Z/A{>}_{wat}}$
	*0.5*	*1*	*2*	*5*		
LiF	0.828(5)	0.830(4)	0.833(3)	0.828(2)	0.833	0.832
Li B O	0.865(5)	0.862(4)	0.869(3)	0.865(2)	0.873	0.874
Diamond	0.879(5)	0.886(4)	0.894(3)	0.888(2)	0.900	0.894
Silicon	0.906(5)	0.907(4)	0.906(3)	0.896(2)	0.894	0.899
Al O	0.883(5)	0.884(4)	0.884(3)	0.873(2)	0.882	0.885
Air	0.883(4)	0.883(4)	0.874(4)	0.885(4)	0.899	0.899

### C. Relative absorbed‐dose energy response correction

#### C.1 125I source

For a given detector, *R* is independent of distance for I125 source. Value of *R* at a given distance for a given material is insensitive to source model and the phantom material. Table 3 presents the values of *R* calculated for selectSeed I125 source in water and bare line I125 source in water, polystyrene and PMMA phantoms for silicon. The results suggest that *R* is insensitive to source model, distance from source and phantom materials. The distance‐independent values of *R* obtained for I125 source (selectSeed or bare I125 source) in water, polystyrene and PMMA phantoms are 1.41, 0.92, 3.97, 0.47, 8.32 and 1.10, respectively, for LiF, ${\text{Li}}_{2}B_{4}O_{7},\;{\text{Al}}_{2}O_{3}$, diamond, Si and air. This suggests that the investigated detectors are good for relative dose measurements in water for I125 sources.

**Table 3:** Monte Carlo‐calculated values of relative absorbed‐dose energy response correction R for silicon detector material for selectSeed I125 source in water phantom and for bare I125 line source in water, polystyrene and PMMA phantoms.

*Distance (along transverse axis of the source) (cm)*	*SelectSeed* I125		*Bare* I125 Line Source	
	*Water*	*Water*	*Polystyrene*	*PMMA*
0.5	8.318	8.324	8.324	8.324
1.0	8.318	8.324	8.324	8.324
2.0	8.318	8.323	8.323	8.323
3.0	8.318	8.322	8.322	8.322
4.0	8.318	8.321	8.321	8.322
5.0	8.317	8.319	8.320	8.321
6.0	8.316	8.319	8.319	8.320
7.0	8.314	8.318	8.319	8.320
8.0	8.314	8.316	8.318	8.319
9.0	8.313	8.315	8.317	8.318
10.0	8.312	8.313	8.317	8.318
11.0	8.310	8.312	8.316	8.317
12.0	8.309	8.310	8.316	8.317
13.0	8.307	8.308	8.316	8.316
14.0	8.306	8.306	8.315	8.315
15.0	8.303	8.304	8.315	8.315

The value of R=1.41 for LiF‐TLD calculated in the present work is consistent with the published value of 1.41 by Meigooni et al.^(^ ^8^ ^)^ Mobit and Badragan^(^ ^22^ ^)^ have also studied the energy response correction for LiF‐TLD micro rods of different diameters. Their study showed that *R* is sensitive to diameter of LiF rod (i.e. the values of *R* are 1.406±0.2% and 1.323±0.2% for rods of 0.1 cm and 0.5 cm diameter (0.6 cm length), respectively.)^(^ ^22^ ^)^ The authors also studied the angular and radial distance dependence of *R*. For a LiF‐TLD of diameter 1 mm calibrated at 1 cm on the transverse axis of the I125 source in water, *R* decreases by a maximum of 3.5% within the 6cm×6cm×6cm calculation region. For the 5 mm diameter LiF‐TLD, *R* decreases by a maximum of 5% in the same region. Note that a 5% uncertainty is assigned to *R* for the LiF TLD‐100 based dosimetry.^(^ ^33^ ^)^

#### C.2 169Yb source

**Figure 3:**
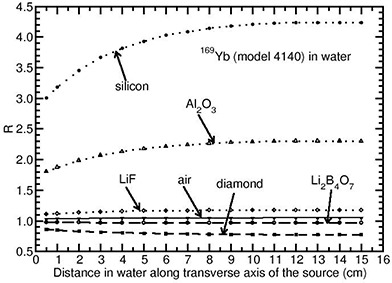
Monte Carlo‐calculated relative absorbed‐dose energy response correction R shown as a function of distance along the transverse axis of the source Yb169 source (model 4140).

Figure 3 presents the Monte Carlo‐calculated values of *R* for the Yb169 (model 4140) source as a function of distance along the transverse axis of the source for different detector materials. For a given detector material, the Yb169 source (model 4140) shows *R* is distance‐dependent (change in *R* is not significant for distances 5 cm and above). For example, *R* increases from 1.11 to 1.18, 1.80 to 2.28, 2.97 to 4.17 and 1.036 to 1.052, respectively for the LiF, ${\text{Al}}_{2}O_{3}$, silicon and air detector materials when the distance is varied from 0.5 cm to 15 cm. This is because mean energy decreases with depth in water (see Fig. 2), which results in increase in *R*. Increase in *R* is substantial for the Si diode detector (up to 40%) as the atomic number is high (see Table I). For the air material, increase in *R* is within 6% as its $Z_{\text{eff}}$ is comparable to that of water. The detector materials, ${\text{Li}}_{2}B_{4}O_{7}$ and diamond have $Z_{\text{eff}}$ values smaller than that of water. Hence, the values of *R* decrease from 0.972 to 0.954 and 0.832 to 0.781, respectively, for the ${\text{Li}}_{2}B_{4}O_{7}$ and diamond detectors, for the above‐mentioned distance range.

MacPherson and Battista^(^ ^15^ ^)^ studied the response for LiF for Yb169 point source as a function of distance in water using the Monte Carlo‐based MCNP code. The values of *R* reported are 1.16 and 1.2 at 1 cm and 4 cm, respectively. In our study, we obtain the values of 1.12 and 1.15 at 1 cm and 4 cm for the bare Yb169 line source, respectively.

**Figure 4:**
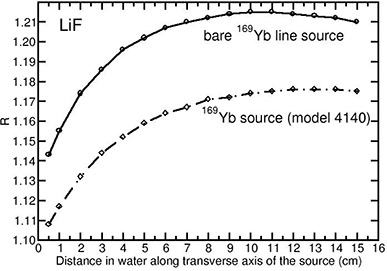
Monte Carlo‐calculated relative absorbed‐dose energy response correction R for LiF for bare line Yb169 source and Yb169 source (model 4140) shown as a function of distance along the transverse axis of the sources.

The Monte Carlo‐calculated values of *R* for the investigated detectors for the bare Yb169 source are different from the Yb169 4140 source model. This is shown in Fig. 4 for the LiF detector. The calculations with the bare Yb169 line source have resulted in overestimation of *R* when compared to the encapsulated Yb169 source (model 4140) for LiF, ${\text{Al}}_{2}O_{3}$ and Si detectors. For example, depending upon the distance, the overestimation is about 3% for LiF (independent of distance), 15% to 22% for silicon, and 11% to 15% for ${\text{Al}}_{2}O_{3}$. For ${\text{Li}}_{2}B_{4}O_{7}$ and air, the variation in the values of *R* between the bare Yb169 source and the encapsulated Yb169 source (model 4140) is only 1%. This difference is comparable to the statistical uncertainty of about 1%. For diamond, the bare Yb169 source has resulted in underestimation of *R* by 5%, independent of distance.

#### C.3 Effect of detector thickness

In general, *R* includes corrections for volume averaging (influence of dose gradients in the detector volume) and self‐absorption by the detector. The energy response of a Scanditronix (IBA Dosimetry GmbH, Schuarzenbruck, Germany) p‐type diode detector (active volume is 2.5 mm diameter and 60 μm thick)^(^ ^12^ ^–^ ^14^ ^,^ ^17^ ^)^ for I125 source has been established to be within ±3.5% for diode‐to‐source distance range of 0.5 cm to 10 cm.^(^ ^12^ ^)^ The published value of average absolute response with respect to dose in water for I125 source is 6.75.^(^ ^12^ ^)^ This value includes self‐attenuation of the diode, which is 0.911.^(^ ^12^ ^)^ The present study gives absolute response value of 7.44 (independent of distance), which does not include self‐attenuation of the diode, because we have not modeled the full diode. When including the published self‐attenuation of 0.911, we obtain average absolute response value of 6.78, which agrees with (within 0.4%) the above‐mentioned published value of 6.75.

In the calculations, we have not modeled the detectors due to limitations associated with the DOSRZnrc and FLURZnrc codes.^(^ ^24^ ^)^ For example, simulation of a cylindrical detector whose axis is parallel to the source axis (cylindrical source) cannot be modeled using the above user‐codes. To quantify approximately the influence of finite detector dimensions on the calculated dose values of I125 and Yb169 sources, we modeled the detectors as cylindrical shells using the DOSRZnrc user‐code. In this study, the Scanditronix p‐type diode detector (60 mm thick active volume of the diode embedded in a circular disk of silicon substrate of diameter 3.5 mm and thickness 0.45 mm)^(^ ^12^ ^–^ ^14^ ^,^ ^17^ ^)^ is modeled as cylindrical shells of height 1 mm. The 60 μm thick sensitive silicon diode (cylindrical shell) material is embedded between 0.225 cm thick silicon substrate shells. Both kerma and absorbed dose are scored in the 60 μm thick sensitive diode region. The values of kerma to Si diode and absorbed dose to Si diode obtained from the DOSRZnrc simulation are statistically indistinguishable. A comparison of dose results obtained from the DOSRZnrc simulations with the FLURZnrc simulation (detector is not modeled in collision kerma calculations) gives self‐attenuation by the diode detector. The value of self‐attenuation by the diode detector obtained for the I125 source is 0.889(1) (independent of distance), which compares reasonably well with the published value of 0.911.^(^ ^12^ ^)^ For the Yb169 source, the values of self‐attenuation by the diode detector obtained at depths of 1 cm, 5 cm, 10 cm and 15 cm in water are 0.992(5), 0.983(5), 0.973(7), and 0.970(7), respectively. A similar study using the 1 mm thick and 1 mm height LiF and ${\text{Al}}_{2}O_{3}$ detectors in water gives negligible self‐attenuation for the Yb169 source at all distances. However, for I125 source, self‐attenuation by LiF is 0.975(5) and by ${\text{Al}}_{2}O_{3}$ is as large as 0.850(1), independent of distance.

#### C.4 Influence of phantom materials on energy response

**Figure 5:**
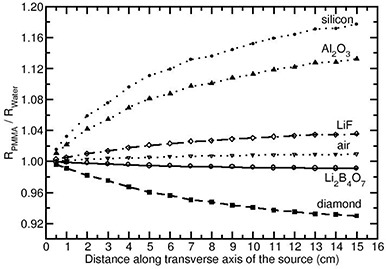
Ratio of the relative absorbed‐dose energy response correction in PMMA phantom to water phantom $R_{\text{PMMA}}$/$R_{\text{Water}}$ presented for different detector materials as a function of distance along the transverse axis of the Yb169 source (model 4140).

**Figure 6:**
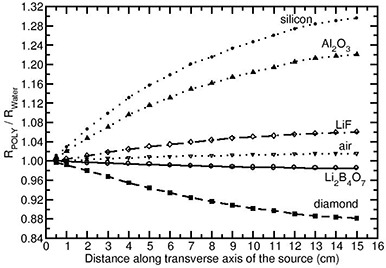
Ratio of the relative absorbed‐dose energy response correction in polystyrene phantom to water phantom $R_{\text{Poly}}$/$R_{\text{Water}}$ presented for different detector materials as a function of distance along the transverse axis of the Yb169 source (model 4140).

The relative absorbed‐dose energy response corrections obtained in the solid phantom materials PMMA, polystyrene and water are designated as $R_{\text{PMMA}},\;R_{\text{Poly}}$, and $R_{\text{Water}}$ respectively. The ratios $R_{\text{PMMA}}$/$R_{\text{Water}}$ and $R_{\text{Poly}}$/$R_{\text{Water}}$ would demonstrate the influence of solid phantoms on the energy response of the detectors compared to the water phantom. Except for the air and ${\text{Li}}_{2}B_{4}O_{7}$ detector materials, the FLURZnrc‐based collision kerma ratios (numerator of Eq. (1)) obtained in the solid phantom materials are different from that in water phantom. Figures 5 and 6 present the values of $R_{\text{PMMA}}$/$R_{\text{Water}}$ and $R_{\text{Poly}}$/$R_{\text{Water}}$ for the Yb169 source (model 4140). These figures demonstrate that both PMAA and polystyrene materials produce similar energy response corrections for air and ${\text{Li}}_{2}B_{4}O_{7}$ detector materials, at all distances. Whereas, for the rest of the detector materials, the values of $R_{\text{PMMA}}$/$R_{\text{Water}}$ and $R_{\text{Poly}}$/$R_{\text{Water}}$ deviate from unity (larger than unity implies over‐response and smaller than unity implies under‐response) as the distance increases. For example, for the Si detector material, the values of $R_{\text{PMMA}}$/$R_{\text{Water}}$ and $R_{\text{Poly}}$/$R_{\text{Water}}$ are 1.18 and 1.30, respectively, at 15 cm depth. For the diamond detector, the values of $R_{\text{PMMA}}$/$R_{\text{Water}}$ and $R_{\text{Poly}}$/$R_{\text{Water}}$ are 0.93 and 0.88, respectively, at 15 cm depth.

## IV. CONCLUSIONS

The Monte Carlo‐based relative absorbed‐dose energy response corrections as a function of depth in water, PMMA and polystyrene phantoms for detector materials such as LiF, ${\text{Li}}_{2}B_{4}O_{7},\;{\text{Al}}_{2}O_{3}$, diamond, silicon and air are calculated for the I125 and Yb169 brachytherapy sources. For the I125 source, the relative absorbed‐dose energy response correction for a given detector is independent of distance in the phantom materials, suggesting that all the detector materials are good for relative dose measurements. For the Yb169 source, the correction is distance‐dependent. As opposed to I125 source, detailed modeling of actual Yb169 is important, as the bare line source‐based response is significantly different from the encapsulated Yb169 source (model 4140). The relative absorbed‐dose energy response of a given detector for the I125 source is insensitive to the phantoms investigated. Whereas, for the Yb169 source, PMMA and polystyrene phantoms demonstrate over‐response for LiF, ${\text{Al}}_{2}O_{3}$ and Si diode and under‐response for diamond when compared to that in water medium. The over‐ or under‐response is significant at large distances. The corrections for the detector materials, air and ${\text{Li}}_{2}B_{4}O_{7}$ are almost identical in all the phantoms.

## ACKNOWLEDGEMENTS

The authors wish to thank Dr. Y.S. Mayya, Head, Radiological Physics and Advisory Division, Bhabha Atomic Research Centre (BARC), Shri. H. S. Khushwaha, Director, Health, Safety and Environment Group, BARC, and Dr. G. Chourasiya, BARC for their encouragement and support throughout this project.
